# Gas-Assisted Spray Fabrication of Reticulated TiO_2_ Scaffolds for Perovskite Solar Applications

**DOI:** 10.3390/mi16060685

**Published:** 2025-06-05

**Authors:** Sana Handor, Andrei Gabriel Tomulescu, Viorica Stancu, Abdelati Razouk, Aurelian Catalin Galca, Lucia Nicoleta Leonat

**Affiliations:** 1 Laboratory of Energetic Engineering and Materials, Science and Technology Faculty, Sultan Moulay Slimane University, Beni Mellal 23000, Morocco; sana.handor@usms.ma (S.H.); a.razouk@usms.ma (A.R.); 2 National Institute of Materials Physics, Atomistilor 405A, 077125 Magurele, Romania; andrei.tomulescu@infim.ro (A.G.T.); stancu@infim.ro (V.S.); ac_galca@infim.ro (A.C.G.)

**Keywords:** spray coating, mesoporous TiO_2_, reticulated structure, perovskite solar cells

## Abstract

This study presents a systematic approach to engineering the electron transport layer (ETL) in perovskite solar cells using a spray deposition technique to fabricate sequentially compact and mesoporous titanium dioxide (c-TiO_2_, m-TiO_2_) films. The spray coating method leads to the development of a distinct reticulated morphology characterized by well-defined wavy-like surface features and significantly increased roughness—at least twice that of spin-coated mesoporous films. The increased interfacial area between the mesoporous TiO_2_ and the perovskite layer facilitates more efficient charge transfer, contributing to higher device performance. By optimizing the deposition parameters, particularly the number of spray cycles for the m-TiO_2_ layer, we achieve a significant enhancement in device performance, with improvements in power conversion efficiency (PCE), reduced series resistance, and minimized hysteresis. Our results demonstrate that an optimal film thickness promotes better perovskite anchoring, while excessive deposition impedes light transmission and increases sheet resistance. These findings advance the practical fabrication of high-performance perovskite solar cells using simple solution-processing techniques and highlights the potential of scalable spray deposition methods for industrial-scale fabrication.

## 1. Introduction

One of the most productive sources of clean electricity relies on harvesting solar energy by means of photovoltaic devices. In addition to the proven silicon technology currently in use, the new branch of perovskite-based solar cells (PSCs) holds great promise in terms of high performance and potentially low production costs [1,2,3]. Since the first report in 2009, hybrid perovskite solar cells’ efficiency has risen from 3.8% to over 25% [4]. Solar cells based on mixed halide perovskites captured considerable interest as a very promising candidate in the photovoltaic field [5,6,7]. These materials are simple to handle and widen the horizons for high-performance and low-cost devices [8,9,10,11].

Nowadays, the main challenge in using PSCs is to bridge the gap between laboratory-scale performance and commercial-scale manufacturing [12,13]. This has driven a shift in focus from conventional deposition techniques toward scalable, reproducible, and industrially viable methods for each layer of the PSC device’s architecture, especially for electron transport layers (ETLs), such as TiO_2_. Spin coating, while ideal for initial research due to its simplicity and uniformity on small substrates, is inherently unsuitable for large-area processing due to significant material waste and difficulty in achieving uniform films over extended areas. Emerging alternatives, such as growth manipulation via electrodeposition techniques [14,15], or other common solution processing techniques, like inkjet printing [16], slot-die coating [17], blade coating [18], and spray coating [19], offer substantial promise in addressing these limitations. These methods support deposition with reduced material usage and compatibility with roll-to-roll processes, which are key requirements for industrial fabrication. Yet, not all component layers can be easily up-scaled, as they are tightly correlated to the deposition technique employed. For example, oxide layers need to maintain critical properties over a large area, such as uniformity, surface adherence, stability, good coverage, crystallinity, and even optical transparency.

Spray coating, and, more specifically, gas-assisted spray coating, is one of the suitable deposition methods for large-area TiO_2_ layers. It enables deposition over large areas with relatively simple equipment, and its operational parameters, such as the nozzle type, gas pressure, substrate temperature, and solution concentration, can be finely tuned to control film morphology and uniformity independent of the type of substrate. Gas-assisted spray coating introduces a pressurized gas stream to atomize the precursor solution, facilitating finer droplet formation and improved substrate coverage.

In terms of material choice for n-type layers in PSCs, common inorganic materials, like metal oxides, and organic materials, such as fullerenes and n-type conjugated polymers, are used as electron transport layers (ETLs). In a mesoscopic n-i-p architecture, they are required to simultaneously fulfill several conditions, including making good contact with the perovskite layer to facilitate electron extraction, exhibit excellent electron transport abilities to the cathode, and provide optical transparency as thin films.

Of all metal oxides, the most versatile is TiO_2_, which was previously used in various different applications from white pigments and coatings due to its high refractive index/brightness and durability [20,21] to electronics as a gate insulator for field-effect transistors (FETs) [22,23]. It has also been used as an electrode material in batteries [24] and in the health and beauty industry due to its low toxicity, such as in sun screens [25,26,27].

Alternative n-type materials like SnO_2_ and ZnO show promise of higher efficiencies and stability due to the higher electron mobility in their bulk single-crystal phases [28]. However, in their nano/mesoporous form, they exhibit different unusual mechanical, electrical, and optical properties as a consequence of their higher surface area exposure. Tiwana et al. [29] measured the effective terahertz mobility of electrons between the nanoporous oxide films and a liquid electrolyte and found mobility values of 0.08 cm^2^/Vs for ZnO, 0.8 cm^2^/Vs for SnO_2_, and 0.1 cm^2^/Vs for TiO_2_. Although the differences between the three oxides are not too far situated, TiO_2_ remains the preferred choice due to its balanced combination of sufficient electron mobility and slower interfacial recombination, which contributes to more efficient charge collection and higher photovoltage compared to SnO_2_ and ZnO. Furthermore, TiO_2_ continues to be a preferred option due to its established performance and compatibility with existing solution manufacturing processes [30,31,32,33].

During the fabrication of mesoscopic n-i-p PSCs, two layers of TiO_2_ are used. The first one is compact, serving as an electron selective layer, and the second is mesoporous to create a high surface area, thus allowing more perovskite material to be deposited and consequently increasing the number of excitons generated upon light absorption. Furthermore, it has been demonstrated that a large specific surface area creates a greater contact interface between the ETL film and the perovskite, thereby improving electron collection [34,35]. Therefore, it is important to control the porosity and thickness of the mesoporous TiO_2_ (m-TiO_2_) film to optimize charge transport and interface stability. These critical parameters are well-controlled when small area devices are fabricated in the laboratory using the ubiquitous spin coating method. However, scaling up to large-area devices requires other equally efficient procedures.

Gas-assisted spray coating supports both compact and mesoporous TiO_2_ layers depending on the formulation and process conditions. Compact layers benefit from the high-speed droplet impingement that promotes dense film formation, while mesoporous layers can be engineered to favor perovskite settlement. Additionally, it was shown that the fabrication of m-TiO_2_ through spray deposition creates a reticulated, crystalline, highly porous surface, which leads to an increased interface length with the perovskite material, thus improving charge collection and the overall efficiency of the solar cells [34], highlighting a key advantage of spray techniques in mesoporous layer deposition [33,36,37,38]. Conversely, the morphology of the compact TiO_2_ (c-TiO_2_) film deposited via spray pyrolysis is almost independent of the number of spray cycles, with the film becoming continuous and compact, partially smoothing and following the profile generated by the FTO.

This research is focused on optimizing the structural, morphological, and optical performances of the m-TiO_2_ films by varying the number of deposition sweeps from 15 to 60, aiming to improve the efficiency of the solar cells with the structure FTO/c-TiO_2_/m-TiO_2_/CH_3_NH_3_PbI_2.6_Cl_0.4_/Spiro-OMeTAD/Au. At the same time, we slightly varied the deposition parameters of c-TiO_2_, doubling the number of sweeps from 15 to 30, to observe if they generate a macro effect on the devices. The overall PCE of all of our devices with mesoporous configuration used in this study is significantly lower than the state-of-the-art values reported in the literature. However, the primary aim of this work is to compare samples with varying degrees of mesoporous reticulation. Consequently, we maintain a consistent material system and concentrate exclusively on analyzing geometric trends and their influence on the device’s efficacy. This work successfully demonstrates the use of the gas-assisted spray deposition method under ambient conditions for the controlled fabrication of both types of films, c-TiO_2_ and m-TiO_2_, without defects, such as cracks or pinholes, achieving full surface coverage and without the use of additives.

## 2. Materials and Methods

### 2.1. Device Fabrication

The FTO-coated glass substrates (15-ohm sq−1 from Xin Yan Technology Ltd., Hong Kong, China) were cleaned with detergent solution and thoroughly rinsed with distilled water, followed by sequential 15 min sonication in acetone (from Chimreactiv SRL, Bucuresti, Romania, 99.5%) and isopropyl alcohol (Chimreactiv SRL, Bucuresti, Romania, 99.9%). The final step was oxygen plasma cleaning for 20 min before usage.

Spray deposition of TiO_2_ was performed using a custom-built in-house system detailed in [33,34]. It employs a Lechler (Metzingen, Germany) pneumatic nozzle (0.4 mm aperture, 45° spray angle) connected to a nitrogen source at 2 kgf/cm^2^ to atomize the precursor solution. The resulting fine mist, with a spray diameter of ~50 mm, was directed at a heated substrate. The setup, which includes computer-controlled nozzle movement in three dimensions, ensures precise and uniform film deposition. Substrates remain fixed on a hot plate throughout the process.

For all devices, the compact TiO_2_ was fabricated through spray pyrolysis using a precursor solution of titanium diisopropoxide bis(acetylacetonate) (from Sigma-Aldrich, Burlington, MA, USA, 75 wt% in isopropanol) that was further diluted by 1:30 vol. in anhydrous isopropyl alcohol (VWR, Radnor, PA, USA, 99.8%). The substrates were placed on a hot plate at 450 °C for at least 20 min prior to the deposition process, and the temperature was kept constant during the deposition. A thermal treatment of 30 min at this temperature was applied to assure crystallization. After the thermal treatment, the substrates were transferred to another hot plate set at 100 °C to lower their temperature naturally.

The reticulated TiO_2_ layers were obtained by spraying a precursor dispersion consisting of a commercial paste of ~20 nm anatase TiO_2_ nanoparticles (from Solaronix T/SP, Aubonne, Switzerland) diluted in anhydrous ethanol (VWR, Radnor, PA, USA, 99.8%) at a mass ratio of 1:200. TiO_2_ mesoporous layers were achieved through 30, 40, 50, or 60 spraying sweeps back and forth. For the samples with spun TiO_2_, we employed the same anatase TiO_2_ paste, although less diluted, at a 1:3 wt ratio at 2000 rpm for 60 s, followed by a drying step at 100 °C for 10 min on the hot plate. After drying and cooling naturally at room temperature, all substrates were placed in a furnace for a thermal treatment of 1 h at 500 °C at a low heating rate of 5 °C/min.

Perovskite layers with the same composition for all devices were fabricated inside of the glove-box through one step of deposition by spin coating a precursor solution consisting of 369 mg of PbI_2_ (Sigma Aldrich, Burlington, MA, USA, 99% purity), 56 mg of PbCl_2_ (Sigma Aldrich; 99.99% purity), and 159 mg of CH_3_NH_3_I (Dyesol, Queanbeyan, Australia). These precursor powders were dissolved in a mixture of 600 mg of DMF (Sigma Aldrich, Burlington, MA, USA, 99.8%) and 78 mg of DMSO (Thermo Scientific, Waltham, MA, USA, 99.9%), resulting in a molar ratio of 8.2:1 of DMF to DMSO. Prior to the deposition, the precursor solution was kept at room temperature in a protected atmosphere with nitrogen while stirring for at least 2 h. While spinning, at 2000 rpm/25 s, right after the first 9 s, 150 μL of DEE (Alfa Aesar, Waltham, MA, USA, 99%) was dropped on it to induce perovskite crystallization. A thermal treatment at 100 °C for 4 min on a hot plate was applied, resulting in a smooth crystalline perovskite film with a CH_3_NH_3_PbI_2.6_Cl_0.4_ (MAPICl) composition.

For the hole transport material (HTM), spiro-OMeTAD was used and prepared as a thin film by spin coating a mixture of 240 mg of spiro-OMeTAD (Borun Chemical, Ningbo, China, 99.8%), 84 μL of 4-tert-butylpyridine (Sigma Aldrich, Burlington, MA, USA, 98%), and 54 μL of bis(trifluoromethane) sulfonimide lithium salt (Alfa Aesar, 98%) in a solution of acetonitrile (Thermo Scientific, 99.8%) at 520 mg/mL, all dissolved in 3 mL of CB (Sigma Aldrich, Burlington, MA, USA, 99.8%). This solution was deposited at 3000 rpm for 60 s in a glove-box at a humidity of less than 20% and a temperature of 20 °C. The samples were left in the glove-box overnight to dry the HTM. Finally, the devices were completed by sputtering 80 nm thick Au electrodes through defined shadow masks with an active area of 0.09 cm^2^ for small-area devices.

### 2.2. Characterization

Atomic Force Microscopy (AFM) images and roughness measurements were recorded using an NT-MDT Aura Ntegra Prima system in noncontact mode. Surface morphology and thickness using cross-sectional images were analyzed with a Carl Zeiss Gemini 500 Scanning Electron Microscope (SEM). Imaging was conducted at an acceleration voltage of 5 kV, a working distance of 9.9 mm, and various magnifications. For device characterization, current–voltage characteristics were measured under AM 1.5 G, and 100 mW cm^2^ simulated sunlight with the Keithley 2000 multimeter was used for real-time DC measurements (electrical resistance or current), being fully computer controlled. The solar simulator was calibrated with an NREL-certified KG5-filtered Si reference diode to an irradiation intensity of 100 mW/cm^2^. The active area of 0.09 cm^2^ was illuminated. Electrical measurements were performed outside, in an ambient environment, at room temperature < 25 °C, and humidity ~50%, and the devices were not encapsulated or protected in any way from the external factors during measurements.

## 3. Results and Discussion

**Figure 1:**
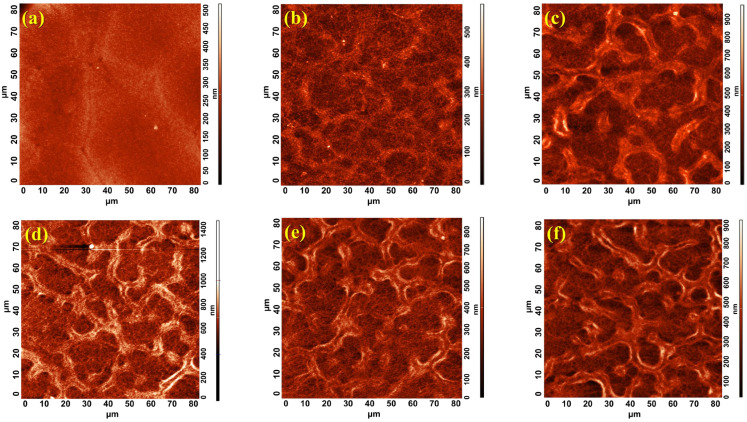
AFM images of 80 × 80 µm scanned area of the m-TiO_2_ made by (**a**) spin coating and spray coating with (**b**) 15x-15x, (**c**) 15x-30x, (**d**) 15x-40x, (**e**) 15x-50x, and (**f**) 15x-60x.

To evaluate the influence of the two methods employed to manufacture the m-TiO_2_ layer, we prepared two different types of samples, including one batch with the spin coating method and the others with the spray coating technique using several sweep cycles, as depicted in Table 1. To understand the impact of these parameters on the film’s roughness, we performed AFM analysis on all of the samples, as shown in Figure 1. A striking difference between the two samples’ surfaces, the spin-coated vs. the spray-coated, is observed. Figure 1a presents the surface of the spin-coated mesoporous layer with the smoothest surface and no pores, whereas in Figure 1b–f the surfaces of the samples with sprayed mesoporous films fabricated using different numbers of spray sweeps present the very distinct formations with random shapes in the form of valleys and hills, with increasingly pronounced formations as the number of spray sweeps increases. These features point to films with complex roughness, which is in fact a specific so-called *reticulated* structure. This structure forms under specific circumstances as the result of the interaction between individual TiO_2_ droplets of the precursor suspension on the hot surface of the samples, a hallmark of the spray deposition technique [33]. The key factors are droplet size, speed, and number, which are in turn influenced by the configuration of the spray system’s elements, such as the nozzle aperture, its distance relative to the substrate, the pressure of the spray gas, and the viscosity of the spray solution, all of which collectively impact the final film’s structure. In contrast, the surface morphology given by spin coating looks almost flat because of the centrifugal force that spreads the solution all over the non-heated substrate. Even though it creates a uniform film on relatively small areas, spin coating may not be ideal for creating larger mesoporous structures.

Table 2 contains roughness parameters calculated from the AFM analysis, which best describe the characteristics of this specific type of structure, including, specifically, the Average Roughness (Sa) and the Root Mean Square (RMS or Sq) corresponding to all of the images shown in Figure 1. Additionally, we used Gwyddion software 2.55 (version 2019-11-04) to calculate the maximum peak height, Sp, which represents the maximum peak height with respect to the mean height calculated from the entire analyzed surface. This parameter is an important measure of surface roughness that quantifies the vertical distance to the highest points on a scanned surface, and, for these samples, it is the only measurement able to characterize the crest of the reticulated structure. Upon analyzing all of these data, there is a clear upward trend of all parameters as the number of sweeps increases, particularly for the thickest mesoporous film.

Analyzing the roughness parameters obtained from AFM measurements, a sharp increase in values is observed from the 15x-15x sample to the 15x-30x sample. This increase is not linear in the subsequent samples, and, when correlated with the images in Figure 1, it provides insight into the degree of formation of the reticulated structure. Thus, at a low number of spray cycles, such as 15x, the reticulated structure does not yet form, even though the substrate is fully covered with mesoporous material. Instead, in this case, a minimum of 30x spray sweeps is required for the formation of the reticulated structure.

On the other hand, increasing the number of sweeps for the compact TiO_2_ film smooths to some extent the substrate’s roughness so that the Sq of the films with 15x-30x compared to the 30x-30x varies only slightly, from 76 to 83 nm. Compared to the sample prepared by spin coating, Sa and Sq increase almost four times for the thickest sample, with TiO_2_ deposited in 15x-60x sweeps, and over fivefold in comparison to the thickest sample, with 30x-60x. These data suggest a direct correlation between the number of sweeps and the thickness of the film obtained for both layers. As the number of cycles increases, the m-TiO_2_ layer becomes thicker both at the bottom of the valleys as well as the height of the valleys’ walls due to the progressive accumulation of material during each pass.

**Figure 2:**
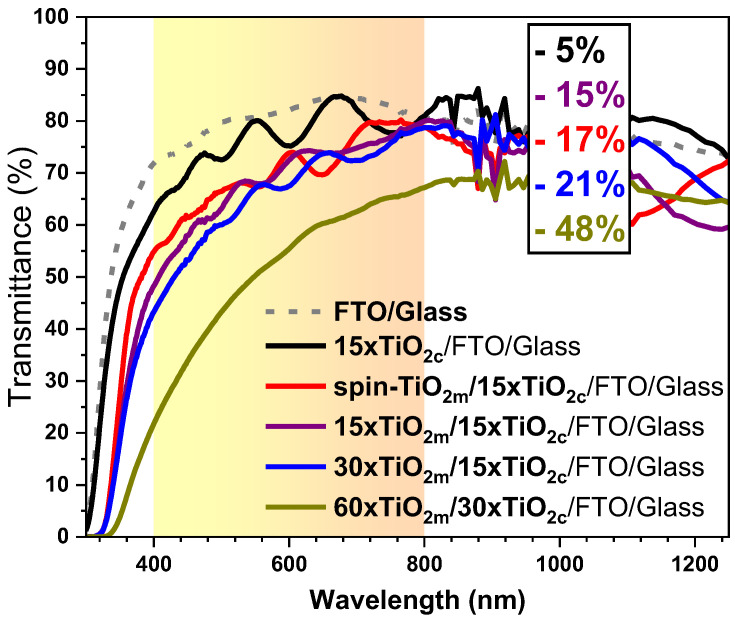
Optical transmittance curves for the samples with different m-TiO_2_.

Yet, too much of the TiO_2_ would interfere with the optical transparency, so, to quantify this unwanted side effect, optical transmission analyses were performed, and the results are presented in Figure 2.

The region of interest, corresponding to the visible spectrum range (400–800 nm) relevant for solar cells, is highlighted in yellow in Figure 2. By integrating the area under the transmission curves in the visible domain, values ranging from ~70% to almost 50% were found that indicate the degree of optical transmittance. Inside of the graph, the percentages of optical loss relative to the FTO/glass substrate are indicated. As expected, samples with fewer deposition cycles exhibited the highest optical transmission, with the best result achieved for samples containing only compact TiO_2_, where only a 5% transmission loss was observed compared with the FTO/glass substrate. The thinnest mesoporous films, corresponding to 15 spray deposition cycles and spin coated, presented 15% and 17% loss, respectively. Increasing the number of spray cycles to 30 generated a transmission of 63% (a 21% loss compared with the FTO/glass substrate), while at 60 cycles the transmission of the sample was only 48%. Most incident light losses are attributed to reflections and scattering at the interfaces between the different films and from the reticulated structure, including the glass substrate’s surface. Additionally, a significant contribution to these losses comes from the FTO coating.

**Figure 3:**
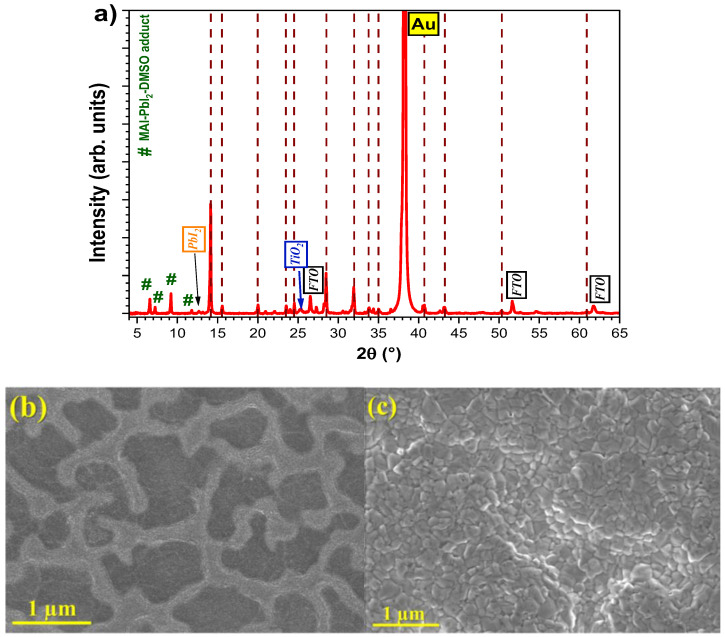
(**a**) XRD pattern of the perovskite film treated with DEE, (**b**) SEM image of the mesoporous surface of the 15x-30x sample, (**c**) SEM image of the perovskite film.

To gain further insights into the influence of the reticulated morphology on the perovskite films’ quality and their consequent effect on the performance of solar cells, perovskite films with the composition CH_3_NH_3_PbI_2.6_Cl_0.4_ were spin coated on top of each type of mesoporous film. Diethyl ether (DEE) was used as an antisolvent to induce crystallization. To assess the quality of the perovskite film, X-ray diffraction (XRD) tests were performed, and surface inspection was carried out using scanning electron microscopy (SEM) both on the mesoporous film itself and after the deposition of the perovskite layer. SEM images were taken on a separate sample, while the XRD was performed directly on a solar cell, as the Spiro-OMeTAD layer does not influence the X-ray diffraction pattern of the hybrid perovskite solar cell. In Figure 3, the XRD pattern and SEM images are shown for a sample with a 15x-30x mesoporous film onto which the perovskite layer was subsequently placed. The diffractogram displayed characteristic crystalline peaks for tetragonally structured perovskite, with characteristic peaks at 14.17°, 15.63°, 20.08°, 28.55°, and 32.05°, according to ICDD 01-084-7607, marked with dashed vertical lines in Figure 3a, and the anatase phase of the n-type TiO_2_ layers at 25.38°, according to ICDD 00-021-1272, as well as for the FTO substrate at 26.53°, 51.69°, and 61.76° (ICDD 00-046-1088) and gold electrodes. Additionally, two secondary phases were observed, one corresponding to PbI_2_ at 12.72°, according to ICDD 00-007-0235, and the other for the intermediate phase of MAI-PbI_2_–DMSO, with characteristic maximums at low angles below 12° [39].

In the SEM image from Figure 3b, a well-defined reticulated structure is visible, where the lighter gray shades represent the higher regions of the structure. The approximate diameters of these valleys (darker gray areas) range from 500 nm to 2 microns. Alternatively, when the perovskite film is deposited, this structure is no longer visible, indicating that a sufficiently thick perovskite layer has been settled over the mesoporous surface. The perovskite film (Figure 3c) appears crystalline, with uniform grains of 100–300 nm in size.

**Figure 4:**
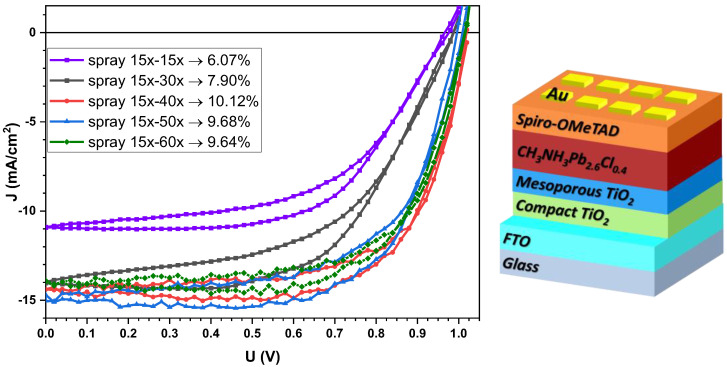
J/V curves of CH_3_NH_3_PbI_2.6_Cl_0.4_ champion solar cells with m-TiO_2_ obtained through different numbers of deposition sweeps and their corresponding PCE. On the right side, the schematic illustration of the structure of the solar cells is shown.

In devices, the photovoltaic characteristics, including Jsc, Voc, Fill Factor (FF), and power conversion efficiency (PCE), vary accordingly, as shown in Figure 4, depicting the champion device curves and their corresponding PCE, and in Table 3, where average parameters from approx. 16 devices of each type are calculated.

Increasing the number of spray sweeps from 15 to 40 results in higher average PCE values (rising from 4.90% to 8.92%) due to the formation of a thicker perovskite film within a more developed reticulated structure (as shown in the AFM images). However, films produced with more than 40 spray coatings form an excessively thick mesoporous layer, which reduces film transparency and limits the number of photons available for absorption by the perovskite, which is in good agreement with the transmittance data presented in Figure 3. For samples with 50x and 60x mesoporous spray coatings, while the champion values remain similar, at 9.68% and 9.64%, the average values show considerable drops from 8.18% to 6.84%, respectively.

Analysis of the data presented in Table 3 revealed different PCE values of devices according to the deposition technique and the spray coating sweep frequency. Spin-coated cells showed a maximum efficiency of 9.82% and an average of 8.61%. In turn, spray-coated cells using variable sweep cycles for the compact and mesoporous layers showed average efficiencies ranging from 4.90% to 8.92% with a maximum of 10.12%. Notably, increasing sweeps of m-TiO_2_ from 15x to 40x almost doubled the average efficiency. Nevertheless, subsequent increases to 15x-50x and 15x-60x resulted in a decrease in efficiency compared to the 15x-40x sweep device.

At first glimpse, in Figure 4, the highest Jsc values are recorded for the samples with centrifuged mesoporous layers. However, this value is not reliable, as other parameters, such as FF, are lower due to high series resistances and low shunt resistances, indicating significant leakage currents, thus reducing the overall efficiency of the devices.

Among all of the devices with spray-deposited mesoporous layers, the worst performance was observed for those with 15x-15x, which exhibited the lowest photocurrent value of only 8.5 mA/cm^2^, which may be a consequence of a thinner perovskite film. Also, changing the number of sprays of the compact TiO_2_ layer from 15x-15x to 15x-30x only slightly improved the cells’ average performance.

This analysis indicates the optimum configuration of spray 15x-40x, giving a champion device with PCE of 10.12%, as represented in Figure 4, with the highest FF of 69% and a low Hi factor. The findings suggest that while initially, increasing the number of sweeps during mesoporous layer deposition enhances efficiency, there seems to be a maximum threshold beyond which additional sweeps can negatively impact the performance of the CH_3_NH_3_PbI_2.6_Cl_0.4_-based solar devices.

**Figure 5:**
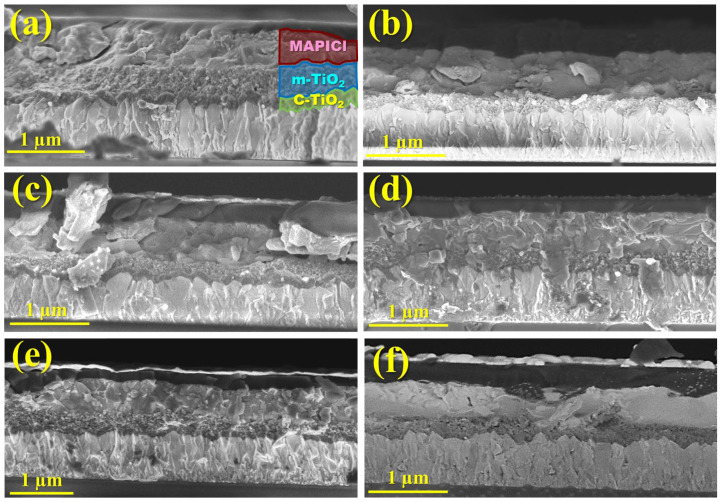
Cross-sectional SEM image of solar cells with different numbers of spray sweeps: (**a**) 15x-spincoated, (**b**) 15x-15x, (**c**) 15x-30x, (**d**) 15x-40x, (**e**) 15x-50x, and (**f**) 15x-60x.

To inspect a correlation between the photovoltaic parameters and the thickness of the TiO_2_ and perovskite films, we fractured one device of each type and visualized these cross-sections using SEM. Figure 5 shows the cross-section SEM images of solar cells with spin-coated and sprayed m-TiO_2_ layers. Thus, while the c-TiO_2_ film, which appears dense, is constant to the entire length and follows the FTO contour with a thickness in the range of 60 to 90 nm, the mesoporous film exhibits varying thicknesses, ranging from a minimum of 70 nm for the sample with 15x-15x to over 490 nm for the sample with 15x-50x, when the cross-sectional cut breaks along a peak from the reticulated structure of the m-TiO_2_. For the sample with the m-TiO_2_ deposited via centrifugation (Figure 5a), the film thickness is clearly constant along the cross-section and exhibits a thickness of approximately 350 nm. For the sprayed layers (Figure 5b–f), these cross-sectional SEM images are of utmost importance, as they allow for the evaluation of the m-TiO_2_ thickness at the bottom of the cavities, observed here at a minimum of ~60 nm to an ~140 nm, whereas AFM images only enable the assessment of the reticulated structure height.

Usually, rougher surfaces disturb the distribution and crystallization behavior of perovskite. Nonetheless, as the reticulated structure becomes more prominent with the increasing number of sweep cycles, the thickness of the perovskite film also increases from ~350 up to over 580 nm, as shown in the SEM cross-section images. This phenomenon occurs due to the improved settlement of perovskite not just on the substrate but also within the walls of the mesoporous cavities.

This research highlights the effectiveness of the spray coating method for producing large-area compact and mesoporous TiO_2_ films, demonstrating the ability of the perovskite films to conform to rough surfaces without affecting solar cell efficiency. By leveraging the advantages of the reticulated structure, this innovative approach paves the way for industrial-scale manufacturing of large-area perovskite solar cells and modules.

## 4. Conclusions

This work demonstrates the critical role of deposition techniques and layer thickness optimization in the performance of perovskite solar cells. TiO_2_ remains a robust and reliable choice for ETLs in perovskite solar cells, offering a balance between efficiency, stability, and ease of fabrication. This study showcases the effective application of gas-assisted spray deposition under ambient conditions to produce both compact (c-TiO_2_) and mesoporous (m-TiO_2_) films with precise control, free from defects like cracks or pinholes and for achieving complete surface coverage without the need for additives. Additionally, the method allows for precise tuning of film thickness and quality simply by adjusting the number of spray passes. Spray fabrication of the m-TiO_2_ layer effectively reduces hysteresis and series resistance, leading to improved FF and ultimately, to higher PCE. The study also emphasizes the importance of balancing the thickness of the TiO_2_ layer, as excessive thickness can impair charge transport and increase resistance. These results highlight the importance of careful control of the manufacturing processes adapted for each component layer to optimize the performance of perovskite solar cells.

## Figures and Tables

**Table 1:** Labels of the fabricated solar devices according to the deposition condition of their TiO_2_ layer.

Sample Label	Deposition of TiO_2_ Layers			
	Compact TiO_2_		Mesoporous TiO_2_	
	Deposition Technique	Number of Sweeps	Deposition Technique	Number of Sweeps
**Spray 15x-spin**	Spray coating	15	Spin coating	--
**Spray 15x-15x**	Spray coating	15	Spray coating	15
**Spray 15x-30x**	Spray coating	15	Spray coating	30
**Spray 15x-40x**	Spray coating	15	Spray coating	40
**Spray 15x-50x**	Spray coating	15	Spray coating	50
**Spray 15x-60x**	Spray coating	15	Spray coating	60
**Spray 30x-30x**	Spray coating	30	Spray coating	30
**Spray 30x-60x**	Spray coating	30	Spray coating	60

**Table 2:** Surface parameters from the AFM analysis.

AFM Parameters FTO/c-TiO_2_/m-TiO_2_	15x-Spin-Coated	Sprayed						
		15x-15x	15x-30x	15x-40x	15x-50x	15x-60x	30x-30x	30x-60x
Average Roughness, Sa (nm)	19	38	60	62	70	77	64	94
Root Mean Square, Sq (nm)	25	49	76	79	88	97	83	116
Maximum peak height, Sp (nm)	194	283	415	423	440	577	429	462

**Table 3:** Photovoltaic parameters of average device values, represented as the average between FW and RW values for multiple solar cells with the same characteristics and an active area of 0.09 cm^2^.

c-TiO_2_ (No. of Dep. Sweeps)	m-TiO_2_ (No. of Dep. Sweeps)	Voc (V)	Jsc (mA/cm^2^)	FF (%)	Rs (Ω cm^2^)	Rsh (Ω cm^2^)	Hi (%)	PCE Average (%)
15	spin-coated	0.998	18.1	48	34	227	4.9	8.61
15	15	0.966	8.5	60	22	2212	4.6	4.90
15	30	0.972	13.0	56	24	1007	4.5	7.03
15	40	0.998	13.4	67	11	1061	2.6	8.92
15	50	0.992	13.4	62	10	710	2.1	8.18
15	60	0.984	10.5	65	13	1415	2.9	6.84
30	30	0.985	12.3	64	9	1111	6.1	7.78
30	60	0.985	13.6	52	25	859	6.2	6.99

## Data Availability

All data are available upon request from the corresponding author.
